# Teaching nursing management of diabetic ketoacidosis: a description of the development of a virtual patient simulation

**DOI:** 10.1186/s41077-022-00241-0

**Published:** 2023-01-11

**Authors:** Fatimazahra Mahou, Saloua Elamari, Adesina Afeez Sulaiman, Oumnia Bouaddi, Omaima Changuiti, Mohammed Mouhaoui, Asmae Khattabi

**Affiliations:** 1 Mohammed VI Center for Research & Innovation, Rabat, Morocco; 2 Mohammed VI University of Health Sciences (UM6SS), Casablanca, Morocco; 3 Faculty of Medicine, Mohammed VI University of Health Sciences (UM6SS), Casablanca, Morocco; 4 Faculty of Nursing Sciences, Ladoke Akintola University of Technology, Ogbomosho, Oyo State Nigeria; 5 College of Health Sciences, International University of Rabat, Rabat, Morocco; 6 Higher Institute of Health Sciences, Laboratory of Health Sciences and Technologies, Hassan First University of Settat, Settat, Morocco; 7 Faculty of Medicine and Pharmacy, Hassan II University, Casablanca, Morocco

## Introduction

Virtual patient simulation (VPS) is an interactive computer simulation that recreates real-world scenarios with the objectives of training, education, and assessment for health care providers [1]. Virtual simulation has been used extensively to adapt nursing education to the COVID-19 pandemic context [2], such as social distancing and/or confinement. The principle of simulation is to allow a better understanding and treatment of common or specific clinical situations that could occur in a real-life context by reproducing them safely and securely [3, 4].

Several comparative studies have shown that distance learning can be utilized as an effective alternative or as a complement to face-to-face training [5] by allowing for knowledge and skills improvement [2, 6]. The results of a study conducted with undergraduate nursing students showed that interactions with virtual patients (VP) can enhance non-technical skills as well [7]. Using VPS, students can make as many mistakes as they can and learn from them [7], which strengthens deep learning, knowledge retention, and learning transfer [8]. During the COVID-19 pandemic, self-esteem, self-confidence, and satisfaction were high among final-year graduate nursing students using virtual simulation [2]. These high levels of satisfaction and self-confidence may be due to the fact that virtual simulation is less stressful because students practice in a safe environment, such as their home, and at their convenience [2, 9]. To make the most of it, simulation-based experience (SBE) must be designed following recognized guidelines [10, 11].

Diabetic ketoacidosis (DKA) is a disorder characterized by uncontrolled metabolic acidosis and excess ketone concentration in the body [12], and it is the most common life-threatening metabolic complication of diabetes especially type I, although it can be present in type II as well [13]. It is worsened by dehydration and acidosis. Considering the severity of DKA and its pattern and frequency of occurrence in patients [14, 15], it is essential that nursing students are versatile with the knowledge of its management in a safe and controlled environment of simulation so as to be able to intervene and manage such cases when in the clinical environment.

This article aims to outline the process of creating a VPS for teaching DKA nursing management using the MedicActiV platform (Bordeaux, France). Several virtual simulations in nursing education have been developed and implemented [16]. However, many gaps remain with regard to the application of instructional design principles, particularly in debriefing practices and the reporting of such practices [16]. In order to address this gap, this article provides step-by-step guidance on the application of the International Nursing Association for Clinical and Simulation Learning (INACSL) Standards of Best Practice for Simulation Design [17, 18, 11, 19].

### INACSL standards of best practice for simulation design

The standards committee is one of 11 INACSL committees that oversee the development of the Healthcare Simulation Standards of Best Practice™ (HSSOBP^TM^), and ensures their quality and upgrade as new evidence and best practices emerge [18]. To provide an effective framework for our virtual patient simulation, we adopted the HSSOBP^TM^ for Simulation Design [11].

In accordance with the HSSOBP^TM^ for Simulation Design [11], 11 criteria were considered in the development of VPS. The following paragraphs show how each criterion was addressed by the VPS developed in this study. It is important to note that the sentences written in *italics* represent the criteria to be respected as they are formulated in the source article.

Criterion 1: “Simulation*-based experiences should be designed in consultation with content experts and simulationists knowledgeable in best practices in simulation education, pedagogy, and practice”.* This virtual SBE was created by a student researcher who completed a postgraduate diploma in simulation for health professions education. Moreover, this SBE is a part of a RCT under the supervision of a healthcare simulation expert.

Criterion 2: “*Perform a needs assessment”*. Several reasons justify the use of DKA nursing management to inform the VPS. Firstly, DKA is one of the most prevalent severe acute complications of diabetes leading to a significant risk of death [20]. The highest rates of DKA are found in low- and middle-income countries, including Morocco [20]. Morocco is one of the 10 countries in the world with the highest estimated incidence and prevalence of type 1 diabetes among children and adolescents aged 0 to 19 years (5.1/1000 and 43.3/1000, respectively) [21]. According to World Health Organization estimates, diabetes kills more than 12,000 Moroccans each year and causes another 32,000 deaths attributed to complications from high blood glucose levels [22]. Secondly, no previous Serious Game (SG) or VPS about this diabetic complication has been developed for healthcare professionals or students in healthcare professions in the French language [23–28]. Thirdly, nurses in different specialties will have to manage a case of DKA at some point during their careers [29]. Thus, the population targeted by the VPS would be of greater size.

Criterion 3: “*Construct measurable objectives”.* A broad goal leads to specific objectives. The general objective of this VPS for nursing students is to learn how to identify and manage DKA. Specific objectives are to

- Assess the patient's clinical condition.
- Consider a DKA diagnosis when there is hyperglycemia.
- Ensure the reception, conditioning, and monitoring of a patient with DKA.
- Begin the nursing management of DKA.
- Provide continuous monitoring to the patient.

Criterion 4: “*Build the simulation-based experience to align the modality with the objectives”*. The simulation modality chosen for this study is a computer-assisted simulation using a VP. To align the modality with the objectives, the VPS phases were designed so that each step requires the student to justify a specific objective. For example, to assess the patient’s clinical condition, the student has to write questions to the VP about his medical history and current symptoms. The conceptual framework chosen to base this SBE is the Jeffries Simulation Framework developed by the National League for Nursing [10, 30]. This SBE includes a starting point, structured learner activities, and an ending point. It begins with a brief briefing on the patient's initial situation. The student then proceeds to the patient interview, the situation pre-analysis, the patient examination, and the final analysis before deciding on a diagnosis. The student then will describe para-clinical examinations and medications and suggest a monitoring strategy and therapeutic education for the patient. The ending point is when scores, duration, and feedback are displayed.

**Fig. 1:**
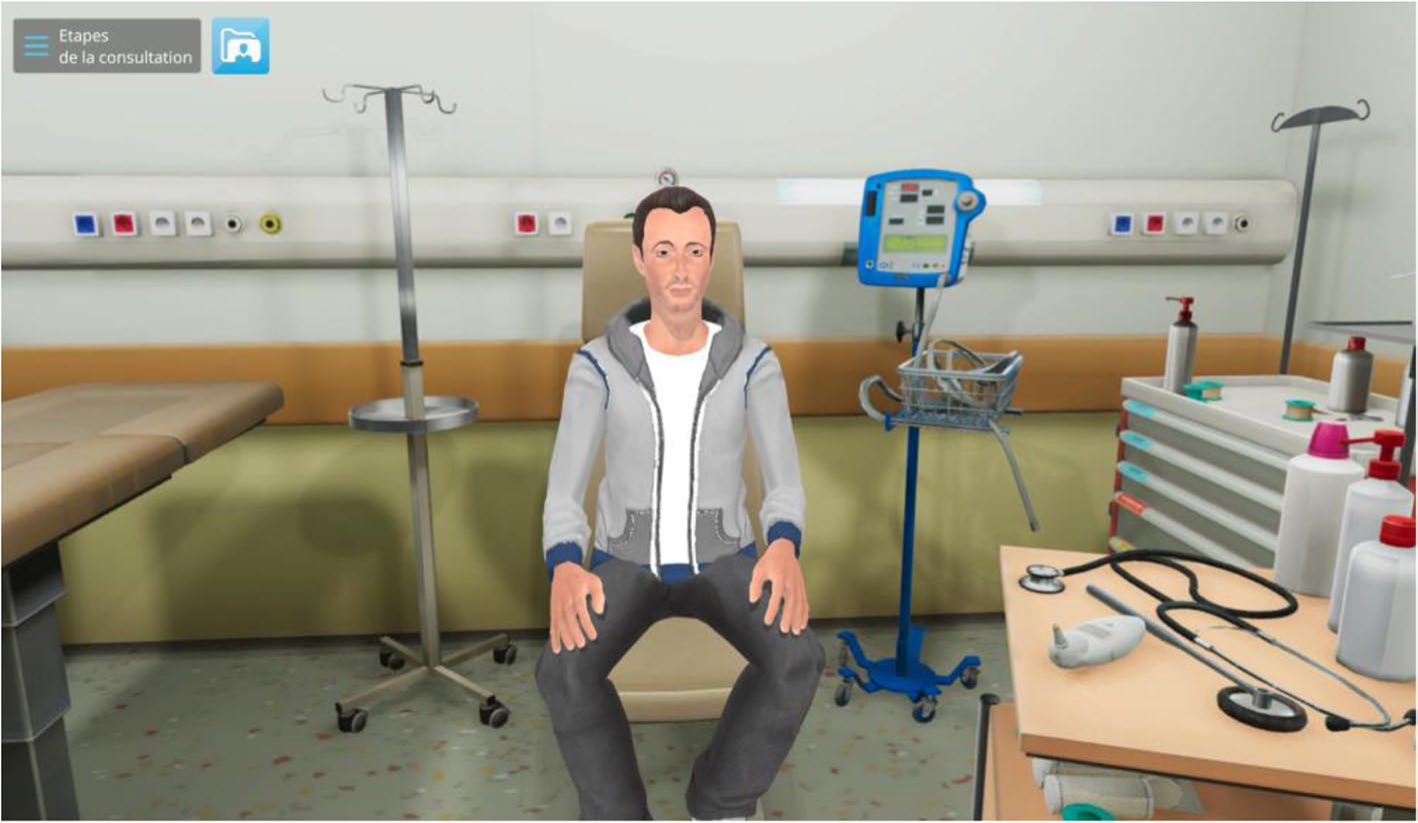
Screenshot of the virtual patient

Criterion 5: “*Design a scenario or case that provides the context”*. A scenario was planned to address the nursing management of DKA. To give a realistic starting point, the backstory of the scenario is about a diabetic 18-year-old single student who suffers from severe stomachache, vomiting, fever, hyperglycemia, bad breath, and missed some insulin injections. Depending on student inquiries, information about the VP’s clinical condition will be provided. Some cues are used to guide the student’s actions such as the weight of the VP who is visibly tired and losing weight (Fig. 1).

Criterion 6: “*Use several types of fidelity (physical, conceptual, and psychological)”.* A degree of fidelity needs to be established according to several factors. First is the learner’s level; an advanced learner may get irritated by a low level of fidelity, whereas a novice learner may be overwhelmed by a high level of fidelity [11, 31, 32]. Since this VPS targets beginners, a medium level of fidelity was authorized. Other factors such as learning objectives, availability, resources, and equipment were considered [11]. Physical fidelity was addressed by creating a virtual environment that mimics reality. This environment presents a 3D VP sitting on a hospital chair in an emergency room wearing a normal outfit that will be removed during the clinical examination. To enhance fidelity, the VP’s body interacts with the student’s actions. For example, when the undergraduate nursing student touches the VP’s chest, a window automatically opens showing what the student should feel or observe in real conditions (like polypnea or chest tenderness on palpation). Conceptual fidelity makes sure that all the scenario’s or case’s components relate to one another realistically so that the learner may understand the patient as a whole. As recommended in the HSSOBP^TM^ for simulation design [11], the scenario is reviewed by experts in healthcare simulation and endocrinology and tested by registered nurses. Psychological fidelity is less assured in this SBE, using only the patient’s-tired voice, the opportunity to interview him, and to hear his responses. Hospital noise and lighting, distractions, family members, other healthcare team members, time pressure, and competing priorities are other psychological elements that mimic real clinical environments and which are not available on this platform. It is worthy of note that defining the fidelity concept in this VPS was constrained by the INACSL standards. Alternative frameworks such as combining functional task alignment with physical resemblance may provide a broader and more realistic understanding of the concept of fidelity and should therefore be recognized [33].

Criterion 7: “*Plan a learner-centered facilitative approach driven by the objectives, learners’ knowledge and level of experience, and the expected outcomes”.* When using the virtual simulation, no trained facilitator is required. The facilitation approach is guaranteed by the various multiple-choice questions throughout the VPS and by the automatic dialogue between the student and his VP. It is worthy of note that this automatically generated dialogue takes place after giving the student the chance to ask the required questions to make a preliminary diagnosis and before the clinical examination.

**Fig. 2:**
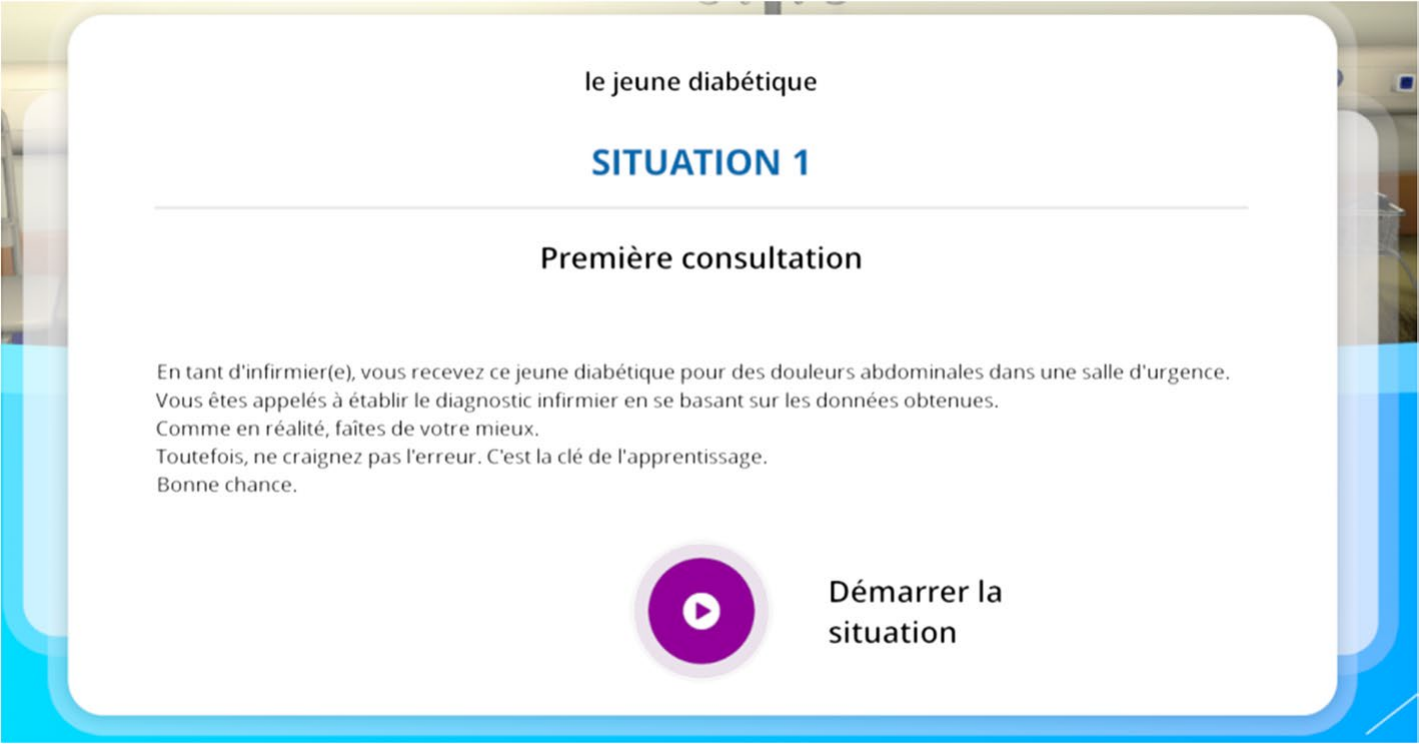
The shortened version of the briefing text

Criterion 8: “*Begin simulation-based experiences with prebriefing”*. Pre-briefing is a two-step process that includes preparation and briefing [34]. The purpose of prebriefing is to make sure that simulation learners are ready for the educational content and are aware of the ground rules for the SBE. In accordance with the HSSOBP^TM^ for prebriefing [34], the prebriefing activities of this simulation were structured as follows:

- Send login and password information for access to the virtual simulation to students via email. The students concerned are those who have fulfilled the requirements (attending endocrinology or medical pathology courses);
- Include in the same email with login information a video recorded by the principal investigator (PI). This video is a step-by-step demonstration of how to use virtual simulation.
- Also send with the login information email a briefing text. Whose main purpose is to establish the students’ psychological safety and clarify the simulation’s educational content. After explaining the VP’s clinical case, information will be provided about what is expected of the student, his or her role, the duration of the simulation (20 to 30 min), the period of authorized free access (one week 24 h a day), source of technical advice, the fictional contract, and assessment methods. The briefing text is written as follows: “You are a nurse working in an emergency department. A diabetic patient named IDRISSI is admitted to your emergency department. He is suffering from severe abdominal pain. Your role is to identify his severe pain etiology and to set up a nursing plan care adapted to his condition. This virtual simulation will take 20 to 30 min, depending on your performance level. For a week, you have unlimited free access to it from anywhere. If technical support is required, please contact the researcher at her e-mail address. Our goal was to create a simulation that was as realistic as we could manage. We want you to behave as you would in real life. This fictional contract does not prevent you from making mistakes. On the contrary, rather than in real life, this is the moment during which you can err the most. As every training must be evaluated, this virtual simulation will be no exception. A written test is scheduled at the end of this experience to assess your knowledge level and evaluate your perceptions and satisfaction with the experience”.
- Once connected to the VPS, the first window will contain a shortened version of the briefing text (Fig. 2) which reminds the student of the simulation’s context and their right to make mistakes.

Criterion 9: “*Follow simulation-based experiences with a debriefing and/or feedback session”*. Following the HSSOBP^TM^ for the debriefing process [35], any of the actions of feedback, debriefing, and/or guided reflection may be included in a debriefing process. Since this SBE is virtual and will be completed on a computer, the debriefing process will be conducted as feedback integrated into the experience as a post-scenario activity. It will address each stage of the virtual simulation, providing the score attained with each question, the correct response, and a brief commentary. However, before they can get feedback, students are notified about their simulation duration and their progress from one simulation to the next.

Criterion 10: “*Include an evaluation of the participant(s), facilitator(s), the simulation-based experience, the facility, and the support team”*. A randomized controlled trial (RCT) is being conducted on this SBE. It begins with a knowledge pre-test and ends with a knowledge post-test and four validated questionnaires that assess student satisfaction, self-confidence, perceptions, and anxiety. Informed consent will be obtained from all participants. Students will be made aware that this SBE is intended to align with the summative evaluation methods in accordance with the HSSOBP^TM^ for Evaluation of Learning and Performance [36]. Scores on all assessments will be reported to students individually at the end of the study.

Criterion 11: *Pilot test simulation-based experiences before full implementation*. The produced SBE will be subjected to acceptance testing in two stages: alpha and beta. It will be alpha tested by its developers and a few graduate nurses. Then, it will be beta tested by students who are similar to the RCT population. This pilot test aims to find any SBE elements that are unclear, lacking, or underdeveloped as well as to guarantee the reliability and validity of all the measurement instruments [37–39]. Any necessary improvements will be made before the actual implementation of the SBE on the RCT population.

## The MedicActiV platform

The virtual platform chosen to implement this virtual SBE is a French platform; MedicActiV which is designed to support digital training for healthcare providers [19]. The MedicActiV platform enables users (e.g., researchers, educators, and healthcare providers) to consult, create, and disseminate simulators. It is possible to use the authoring tool and test the demonstration simulators by creating a free account. In addition, the MedicActiV platform allows users to add other users, manage simulators, create training sessions, or track learners’ outcomes. The MedicActiV Store can be used to buy simulators designed by authors from around the world or to sell simulators designed independently or as part of a project with SimforHealth.

In this section, we describe how the MedicActiV platform was used to design a clinical case on the nursing management of DKA.

An engineer from the MedicActiV team created an account for the PI. Once logged into the platform, the writing tool was downloaded and completed by the PI. Four sections make up the authoring tool: simulator data, patient interview settings, consultation information (including a patient avatar, consultation setting and steps, synthesis, and appendices), and author information.

### Simulator data

In this section, we provided the virtual simulator title, its country, its specialty, the target population, and its training level (beginner, intermediate, or advanced if initial training, experienced if continuous training). In this part, we added a summary of the scenario, the number of consultations, and VPS pedagogical goals.

### Patient interview settings

There are two ways to play out the interview on the MedicActiV platform: an interview with a dialogue simulator or an interview with preprogrammed questions. In the first format, students can type questions for the VP to answer, and the instructor must carefully prepare the patient’s medical file while completing the authoring tool. In the second setting, learners will have to choose from a list of questions that the instructor has already defined, and then they will hear the answer from the VP. For this mode, the questions and answers must be written by the instructor when filling in the authoring tool. In this DKA clinical case, an interview with a dialogue simulator is the chosen option.

### Consultation information

The first consultation is the learner’s first contact with the VP. It is at this time when the patient’s avatar, the patient’s medical file, the consultation information and setting, and the consultation stages are set up. A synthesis and appendices are added at the end of each consultation.

To program the patient’s avatar, information such as his gender, age, weight, ethnicity, whether he wears clothes or not while the clinical examination, his voice depending on his age (old or young), and his health status (normal, tired, or worried) was provided.

The patient’s medical file comprises the patient’s information that was known before the consultation. It is updated according to the data gathered as the consultation progresses. As a result, a patient’s history is established for the following sub-folders: the reason for the consultation, the history of the disease, the patient's history (medical, family, and surgical), lifestyle, current treatments, and results of any additional tests. The instructor can select which medical record sub-folders he wishes to fill in for the consultation, but those marked with an asterisk (*) are required. One example is the patient profile sub-folder, which includes the patient’s name, gender, age, career, marital status, number of children, age of children, height, weight, and abdominal diameter.

The consultation’s information includes the title, an abridged context, and the date. At this point, the instructor has the option of deciding whether the patient is present for the consultation or not. The “Consultation without patient” option is available for a consultation that consists solely of an examination of test findings, an analysis of a medical report, a telephone interview with the patient, and so on.

Three consultation settings are available on the authoring tool: hospital, doctor’s office, or patient’s residence. However, the platform presents more diverse and specific settings.

**Fig. 3:**
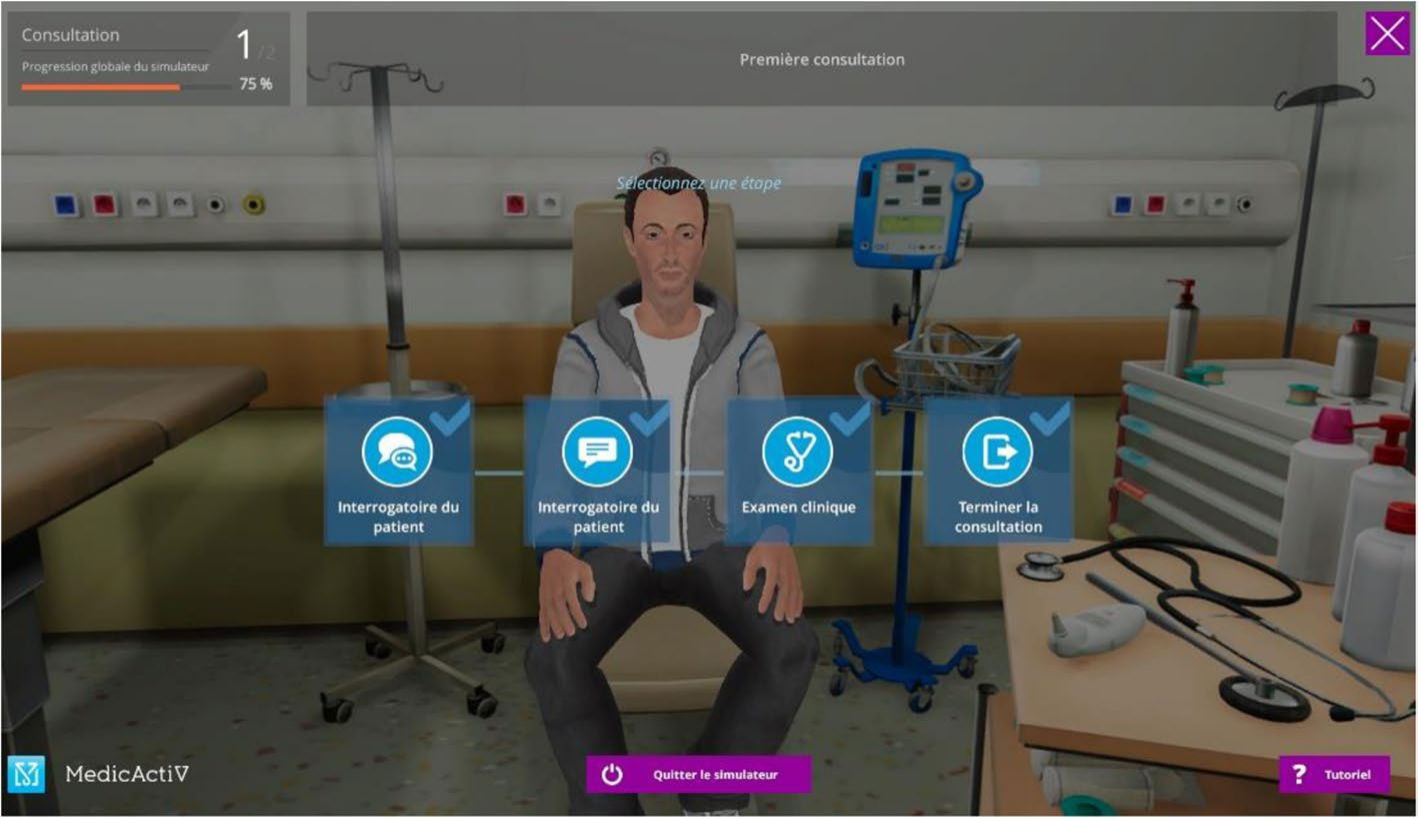
The diabetic ketoacidosis scenario steps

**Fig. 4:**
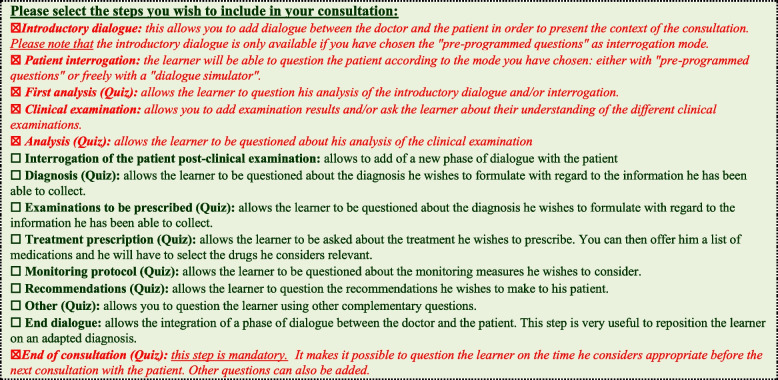
The full steps of a consultation

The consultation is organized in steps. The instructor must choose the steps that will make up his scenario. The learner will find these steps on the menu at the top left corner. The learner must then respect the order of the steps (Fig. 3). The steps marked “Quiz” allow the learner to be questioned through one or more questions (Fig. 4). These questions can be multiple-choice, single-choice, or open questions.

Figure 4 shows all the steps that can be included in a consultation. The instructor filled out only the ones they have selected. The steps used to develop the nursing management of DKA scenario are in *italics and red*.

Each consultation concludes with a synthesis and appendices. The synthesis summarizes in a maximum of 1000 characters the key elements of the consultation. The student can then enhance his or her understanding of the topic by consulting the documents included in the appendices, such as suggestions, research, or other scientific documents. The appendices might include text, images, videos, website links, PDF documents, and more.

### Authors’ information

At the end of the authoring tool, the instructor entered his title, full name, and a brief self-introduction, as well as acknowledged any conflicts of interest.

After informing the authoring tool, the next step was to implement it on the platform. It is essential to mention that the process in the platform is organized the same way as the authoring tool. The MedicActiV platform allowed the PI to preview his VPS before sharing it with students who had already provided their email addresses.

## Conclusion

In the past few years, the use of simulation has supplemented traditional nursing education [40]. This study outlined the creation of a VPS about DKA by incorporating the HSSOBP^TM^ for Simulation Design and the MedicActiV platform. The HSSOBP^TM^ criteria, as well as those of MedicActiV, were discussed. They illustrate the value of virtual simulation in teaching the assessment and management of patients, including those with DKA. VPS provides a safe and controlled environment where nursing students can translate their theoretical knowledge into skills and practice.

## Data Availability

The datasets used and/or analyzed during the current study are available from the corresponding author upon reasonable request.

## Abbreviations

DKA: Diabetic ketoacidosis
HSSOBP^TM^: Healthcare Simulation Standards of Best Practice^TM^
INACSL: International Nursing Association for Clinical and Simulation Learning
PI: Principal investigator
RCT: Randomized controlled trial
SBE: Simulation-based experience
VP: Virtual patient
VPS: Virtual patient simulation

## References

[1] Rouleau G, Pelletier J, Côté J, Gagnon M, Martel-Laferrière V, Lévesque R, SimforHealth, Fontaine G. Codeveloping a Virtual Patient Simulation to Foster Nurses’ Relational Skills Consistent With Motivational Interviewing: A Situation of Antiretroviral Therapy Nonadherence. J Med Internet Res. 2020;22(7):e18225. https://www.jmir.org/2020/7/e18225. 10.2196/18225 PMC7391166 32672679

[2] Zaragoza-García I, Ortuño-Soriano I, Posada-Moreno P, Sánchez-Gómez R, Torredà M (2021). Virtual simulation for last-year nursing graduate students in times of Covid-19: a quasi-experimental study. Clin Simul Nurs..

[3] Cant RP, Cooper SJ (2017). Use of simulation-based learning in undergraduate nurse education: an umbrella systematic review. Nurse Educ Today..

[4] Alamrani MH, Alammar KA, Alqahtani SS, Salem OA (2018). Comparing the effects of simulation-based and traditional teaching methods on the critical thinking abilities and self-confidence of nursing students. J Nurs Res..

[5] Newman NA, Lattouf OM (2020). Coalition for medical education—a call to action: a proposition to adapt clinical medical education to meet the needs of students and other healthcare learners during COVID-19. J Card Surg..

[6] Goldsworthy S, Muir N, Baron S, Button D, Goodhand K, Hunter S (2022). The impact of virtual simulation on the recognition and response to the rapidly deteriorating patient among undergraduate nursing students. Nurse Educ Today..

[7] Peddle M, Bearman M, Mckenna L, Nestel D (2019). Exploring undergraduate nursing student interactions with virtual patients to develop ‘non-technical skills’ through case study methodology. Adv Simul.

[8] King A, Holder MG, Ahmed RA (2013). Errors as allies: error management training in health professions education. BMJ Qual Saf..

[9] Kang SJ, Hong CM, Lee H (2020). The impact of virtual simulation on critical thinking and self-directed learning ability of nursing students. Clin Simul Nurs..

[10] al Khasawneh E, Arulappan J, Natarajan JR, Raman S, Isac C. Efficacy of simulation using NLN/Jeffries Nursing Education Simulation Framework on satisfaction and self-confidence of undergraduate nursing students in a middle-eastern country. SAGE Open Nurs. 2021;7. Available from: https://pubmed.ncbi.nlm.nih.gov/33959680/ cited 2022 Sep 28 10.1177/23779608211011316 PMC8060750 33959680

[11] Watts PI, McDermott DS, Alinier G, Charnetski M, Ludlow J, Horsley E (2021). Healthcare Simulation Standards of Best PracticeTM simulation design. Clin Simul Nurs..

[12] Lizzo JM, Goyal A, Gupta V. Adult diabetic ketoacidosis. StatPearls. 2022. Available from: https://www.ncbi.nlm.nih.gov/books/NBK560723/. cited 2022 Oct 15 32809558

[13] Getie A, Wondmieneh A, Bimerew M, Gedefaw G, Demis A (2021). Determinants of diabetes ketoacidosis among diabetes mellitus patients at North Wollo and Waghimra zone public hospitals, Amhara region, Northern Ethiopia. BMC Endocr Disord..

[14] al Hayek AA, al Dawish MA (2021). Frequency of diabetic ketoacidosis in patients with type 1 diabetes using FreeStyle Libre: a retrospective Chart Review. Adv Ther..

[15] Davis TME, Davis W (2020). Incidence and associates of diabetic ketoacidosis in a community-based cohort: the Fremantle Diabetes Study Phase II. BMJ Open Diabetes Res Care.

[16] Foronda CL, Fernandez-Burgos M, Nadeau C, Kelley CN, Henry MN (2020). Virtual simulation in nursing education: a systematic review spanning 1996 to 2018. Simul Healthc..

[17] About INACSL. Available from: https://www.inacsl.org/about-inacsl. cited 2022 Sep 28

[18] Committees. Available from: https://www.inacsl.org/committees. cited 2022 Sep 28

[19] MedicActiV | La plateforme de formation des professionnels de santé par simulation numérique. Available from: https://www.medicactiv.com/fr/. cited 2022 Sep 28

[20] IDF Diabetes Atlas 10th edition; Available from: www.diabetesatlas.org. cited 2022 Jun 7

[21] LRhys Williams (Chair) SC (Deputy C, Basit DB, Bright JC, Edward Gregg LG, Motala GO, Pearson-Stuttard AR, et al. IDF DIABETES ATLAS. 2020. Available from: https://www.diabetesatlas.org/upload/resources/material/20200302_133351_IDFATLAS9e-final-web.pdf. cited 2022 Sep 28

[22] WHO EMRO | Journée mondiale de la Santé : ensemble contre le diabète | Actualités | Maroc. Available from: http://www.emro.who.int/fr/mor/morocco-news/journee-mondiale-de-la-sante-ensemble-contre-le-diabete.html. cited 2022 Jun 7

[23] Peddle M, Bearman M, Nestel D. Virtual patients and nontechnical skills in undergraduate health professional education: an integrative review. Clin Simul Nurs. 2016;12(9):400–10.

[24] Cook DA, Erwin PJ, Triola MM (2010). Computerized virtual patients in health professions education: a systematic review and meta-analysis. Acad Med..

[25] Kononowicz AA, Woodham LA, Edelbring S, Stathakarou N, Davies D, Saxena N (2019). Virtual patient simulations in health professions education: systematic review and meta-analysis by the digital health education collaboration. J Med Internet Res.

[26] Malicki A, Vergara FH, van de Castle B, Goyeneche P, Mann S, Scott MP (2020). Gamification in nursing education: an integrative literature review. J Contin Educ Nurs..

[27] Haoran G, Bazakidi E, Zary N. Serious Games in Health Professions Education: Review of Trends and Learning Efficacy. Yearb Med Inform [Internet]. 2019;28(1):240–8. [cited 2023 Jan 6]. Available from: http://www.thieme-connect.de/products/ejournals/html/10.1055/s-0039-1677904. 10.1055/s-0039-1677904 PMC6697512 31022747

[28] Shorey S, Ng ED (2021). The use of virtual reality simulation among nursing students and registered nurses: a systematic review. Nurse Educ Today..

[29] Volkova NB, Fletcher CC, Tevendale RW, Munyaradzi SM, Elliot S, Peterson MW. Impact of a multidisciplinary approach to guideline implementation in diabetic ketoacidosis. 2008;23(1):47–55. Available from: 10.1177/1062860607311015?journalCode=ajmb. cited 2022 Sep 28 10.1177/1062860607311015 18187590

[30] Ravert P, McAfooes J (2014). NLN/Jeffries Simulation Framework: State of the science summary. Clin Simul Nurs..

[31] Johnsen HM, Fossum M, Vivekananda-Schmidt P, Fruhling A, Slettebø Å (2018). Nursing students’ perceptions of a video-based serious game’s educational value: a pilot study. Nurse Educ Today..

[32] Singh D, Kojima T, Gurnaney H, Deutsch ES. Do fellows and faculty share the same perception of simulation fidelity? A pilot study. simulation in healthcare. 2020;15(4):266–70. Available from: https://journals.lww.com/simulationinhealthcare/Fulltext/2020/08000/Do_Fellows_and_Faculty_Share_the_Same_Perception.7.aspx. cited 2022 Sep 28 10.1097/SIH.0000000000000454 32371750

[33] Hamstra SJ, Brydges R, Hatala R, Zendejas B, Cook DA (2014). Reconsidering fidelity in simulation-based training. Acad Med..

[34] McDermott DS, Ludlow J, Horsley E, Meakim C (2021). Healthcare simulation standards of Best PracticeTM prebriefing: preparation and briefing. Clin Simul Nurs..

[35] Decker S, Alinier G, Crawford SB, Gordon RM, Jenkins D, Wilson C (2021). Healthcare Simulation Standards of Best PracticeTM the debriefing process. Clin Simul Nurs..

[36] McMahon E, Jimenez FA, Lawrence K, Victor J (2021). Healthcare Simulation Standards of Best PracticeTM evaluation of learning and performance. Clin Simul Nurs..

[37] van Teijlingen E, Hundley V (2002). The importance of pilot studies. Nurs Stand..

[38] Devon HA, Block ME, Moyle-Wright P, Ernst DM, Hayden SJ, Lazzara DJ (2007). A psychometric toolbox for testing validity and reliability. J Nurs Scholarsh..

[39] Coupaud M, Castéra J, Cheneval-Armand H, Brandt-Pomares P, Delserieys A. Développer un questionnaire pour étudier les conceptions de l’évolution du vivant d’élèves de collège : entre cadres didactique et psychométrique. http://journals.openedition.org/rdst. 2019 Dec 31 [cited 2022 Sep 20];(20):27–59. Available from: http://journals.openedition.org/rdst/2641

[40] Changuiti O, Moustarhfir N, Marfak A, Saad E, Hilali A, Youlyouz-Marfak I (2021). Simulation based-learning from simple to complicated clinical situations for midwifery students. Adv Med Educ Pract..

